# Distribution of axial length in Japanese children and adolescents aged 4 to 19 years

**DOI:** 10.1007/s10384-026-01328-1

**Published:** 2026-01-19

**Authors:** Shunsuke Fujioka, Naoko Takada, Sayaka Yoshida, Mami Ishikuro, Masayuki Kobayashi, Genki Shinoda, Aoi Noda, Masatsugu Orui, Taku Obara, Satoru Tsuda, Noriko Himori, Akiko Hanyuda, Ryo Kawasaki, Shinichi Kuriyama, Nobuo Fuse, Toru Nakazawa

**Affiliations:** 1 Department of Ophthalmology, Tohoku University Graduate School of Medicine, 1-1 Seiryo-machi, Aoba-ku, Sendai, Miyagi 980-8574 Japan; 2 Department of Preventive Medicine and Epidemiology, Tohoku Medical Megabank Organization, Tohoku University, 2-1 Seiryo-machi, Aoba-ku, Sendai, Miyagi 980-8573 Japan; 3 Division of Molecular Epidemiology, Tohoku University Graduate School of Medicine, 2-1 Seiryo-machi, Aoba-ku, Sendai, Miyagi 980-8573 Japan; 4 School of Medicine, Tohoku University, 2-1 Seiryo-machi, Aoba-ku, Sendai, Miyagi 980-8574 Japan; 5 International Research Institute of Disaster Science, Tohoku University, 2-1 Seiryo-machi, Aoba-ku, Sendai, Miyagi 980-8573 Japan; 6 Division of Ophthalmic Precision Medicine Development, United Centers for Advanced Research and Translational Medicine (ART), Tohoku University Graduate School of Medicine, Seiryo-machi, Aoba-ku, Sendai, Miyagi 980-8575 Japan; 7 Department of Advanced Ophthalmic Medicine, Tohoku University Graduate School of Medicine, Seiryo-machi, Aoba-ku, Sendai, Miyagi 980-8574 Japan; 8 Department of Ophthalmology, Keio University School of Medicine, 35 Shinanomachi, Shinjuku‑ku, Tokyo, 160‑8582 Japan; 9 Department of Social Medicine (Public Health), Graduate School of Medicine, University of Osaka, 2-2 Yamadaoka, Suita, Osaka 565-0871 Japan; 10 Department of Integrative Genomics, Tohoku Medical Megabank Organization, Tohoku University, 2-1 Seiryo-machi, Aoba-ku, Sendai, Miyagi 980-8573 Japan; 11 Department of Retinal Disease Control, Tohoku University Graduate School of Medicine, Seiryo-machi, Aoba-ku, Sendai, Miyagi 980-8574 Japan

**Keywords:** Axial length, Myopia, Japanese children, Cross-sectional study

## Abstract

**Purpose:**

To investigate the distribution of axial length (AL) and the prevalence of long axial length (LAL) in Japanese children and adolescents.

**Study design:**

cross-sectional observational study

**Methods:**

We analyzed AL data from 14,482 participants (7,457 boys and 7,025 girls) aged 4–19 in the Tohoku Medical Megabank Project Birth and Three-Generation Cohort Study in Japan. AL was measured using a non-contact optical axis measurement device. We evaluated the distribution of AL in the participants with box plots for age. Segmented regression identified age-related trends and breakpoints. We calculated the age-specific proportions of participants with AL ≥ 24.5 mm and those with AL ≥ 26 mm, defined as LAL, which were considered indicative of suspected myopia.

**Results:**

Mean AL increased with age, with a break point at 11.73 years. The slope before the break point was β = 0.27, while the slope after the break point decreased to β = 0.12. Boys showed earlier break point (9.87 years) than girls (15.91 years). Proportions with AL ≥ 24.5mm and ≥26mm began to increase approximately at 8 and 10 years of age respectively, with sex differences noted between ages 7 to 10 years and 10 to 11 years.

**Conclusion:**

This was the first large-scale AL survey in Japan and revealed the age and sex related distribution of AL and the proportion with LAL among Japanese children and adolescents aged 4 to 19 years.

**Supplementary Information:**

The online version contains supplementary material available at 10.1007/s10384-026-01328-1.

## Introduction

In 2015, the World Health Organization and the Brien Holden Vision Institute issued a report warning of the rapid increase in myopia, calling attention to myopia as a public health problem due to the high risk of visual impairment [1]. Holden et al. performed a meta-analysis that found that the global prevalence of myopia (≤ -0.50 diopter [D]) had been 22.9% in 2000; they predicted that it would increase to 49.8% in 2050, leading to a rise in the prevalence of high myopia (defined in the study as ≤−5.00 D) from 2.7% (2000) to 9.8% (2050) [2]. In Southeast Asian countries, the rate of myopia has increased from 20% to 80% over the past 60 years, and in China, 90% of people in their 10s to 20s are myopic [3]. On the other hand, an epidemiological study in the United States reports that the rate of myopia had increased from 25% to 42% over 30 years [4]; thus, the increase in myopia is considered to be most serious in East and Southeast Asia, including Japan.

High myopia, defined as a spherical equivalent (SE) power of -6.00 D or less [5], generally corresponds to an axial length (AL) of 26 mm or more and significantly increases the risk of serious complications later in life, such as myopic maculopathy, retinal detachment, and glaucoma [6–8]. Appropriate evaluation and management of myopia are necessary to prevent its progression, especially in children. Myopia is evaluated using SE; however, measuring refractive power in children is challenging because their subjective refractive power tends to fluctuate. Even objective refractive measurements can be difficult to interpret due to subtle changes caused by accommodative interventions. Additionally, it is impractical to routinely administer cycloplegic eye drops to eliminate these accommodative effects. It is reported that there is a strong correlation between SE and AL, and that AL increases as myopia progresses [9]. Since around 2000, non-contact optical AL measurement has been introduced, making it easy to measure AL, even in children. Non-contact optical AL measuring devices have smaller measurement errors than contact-type devices, even when used by different examiners, making them suitable for estimating the onset and progression of myopia in young people [10].

To date, there have been reports on AL in young people with a large number of participants from East Asia, mainly China and Europe [11, 12]. However, there is still no large-scale database for AL in young Japanese. Okabe et al. report on AL in 399 children aged 8 years [13], Itoi et al. report on AL in 182 young people aged 7 to 21 years [14], and Terasaki et al. report on AL in 122 children aged 8 to 9 years [15], but a larger-scale report in Japan is currently needed. Regarding younger children, aged 6 years and younger, there is a report by Matsumura et al. on AL in 457 children aged 4 to 6 years in Japan [16], but there are few reports worldwide.

The purpose of this study was to investigate the distribution of AL in Japanese children and adolescents aged between 4 and 19 years and the proportion with long axial length (LAL) in data obtained from the Tohoku Medical Megabank's large-scale cohort study.

## Subjects and Methods

The Tohoku Medical Megabank Project Birth and Three-Generation Cohort Study (TMM BirThree Cohort Study) is conducted in Miyagi Prefecture, Japan. Pregnant women and, later, their children, who lived in Miyagi Prefecture or targeted areas of Iwate Prefecture were recruited between 2013 and 2017. Following recruitment of the women and their children to the TMM BirThree Cohort Study, the children’s siblings, fathers, grandparents, and other family members were also recruited. All participants were included after providing full written informed consent in accordance with the Declaration of Helsinki on Medical Research Involving Human Subjects. The TMM BirThree Cohort Study was approved by the internal review board of the Tohoku Medical Megabank Organization, Tohoku University (No. 2013-1-103-1). The details of the TMM BirThree Cohort Study are described elsewhere [17, 18].

**Fig 1:**
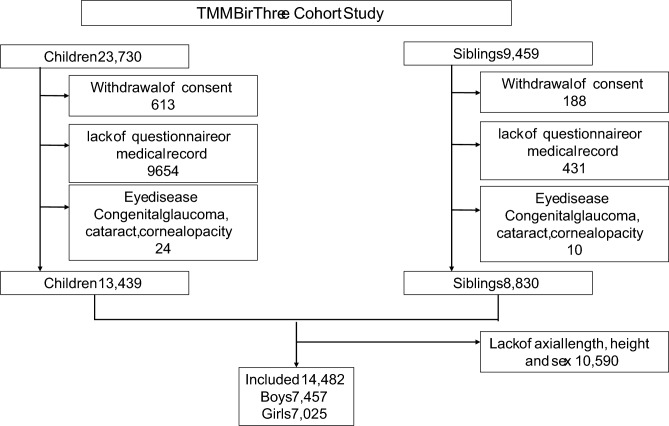
Research flow. Research flow; 14,482 children were included. TMM BirThree Cohort Study; Tohoku Medical Megabank Project Birth and Three-Generation Cohort Study

During the follow-up period, all participants aged 4 years or older were invited to the research facilities to provide physiological assessments, including their AL, in what was collectively known as the ToMMo Eye Study [19]. Until now, participants were invited once or twice according to their ages (aged 4-7 years, 8-15 years, 16-19 years). The invitation for the 4–7-year-old survey is first sent at age 4, and the invitation for the 8–15-year-old survey is first sent at age 8. A total of 2,801 participants provided two measurements. Due to the characteristics of the cohort enrollment, the second measurement was conducted among participants aged 8 years or older and measurements were more frequently obtained at ages 4 and 8. The data for children aged > 8 years are derived only from the siblings’ category of the TMM BirThree Cohort Study. This study reports the distribution of AL in children and adolescents, as well as their siblings, measured between June 2017 and March 2023. After excluding children and siblings who have withdrawn their consent, those whose questionnaires or medical records were missing, those who had ophthalmological diseases that could affect measurement values, or whose height and sex were missing, the data on AL for 14,482 children and their siblings were available (Fig. 1).

AL was measured with the OA-1000 or OA-2000 Optical Biometer (Tomey). A genome medical research coordinator and a licensed nurse or medical technologist measured AL in the participants 10 times, and the data were averaged for each eye, both right and left. The ages of the children and their siblings were calculated according to the date of the measurement and the date of birth.

The distribution of AL in both boys and girls is shown as box plots by age. In order to evaluate changes in the degree of elongation of AL, we used segmented regression to determine the break points for mean and variance in AL by age. The mean and standard deviation of AL, as well as percentiles by age and sex, were also determined and compared with previously reported international data [11, 12]. The t-test was used to evaluate differences in AL between boys and girls in each age group. A paired t-test was used to evaluate differences in AL between right and left eyes in each age group (a threshold p-value with Bonferroni correction of < 0.05/16 [almost equal to 0.0031] was used to determine statistical significance). There is no universal consensus for defining myopia severity by AL, and the reference values for AL vary with age. Previous reports demonstrate a correlation between refractive error and AL, indicating that the degree of myopia can be reasonably estimated from AL [7–9, 20]. In Japan, Tokoro et al., in a report commissioned by the Ministry of Health and Welfare, defined pathologic myopia as ≥24.5 mm at ages 6–8 years and ≥26.0 mm at ages 9–12 years [21]. Therefore, we calculated the proportions of individuals with AL ≥24.5 mm and ≥26.0 mm, defined as LAL, to evaluate the prevalence of suspected myopia, and examined sex difference using Fisher's exact test. SAS version 9.4 (SAS Institute) and R version 4.1.2 were used to calculate statistics.

## Results

**Fig. 2:**
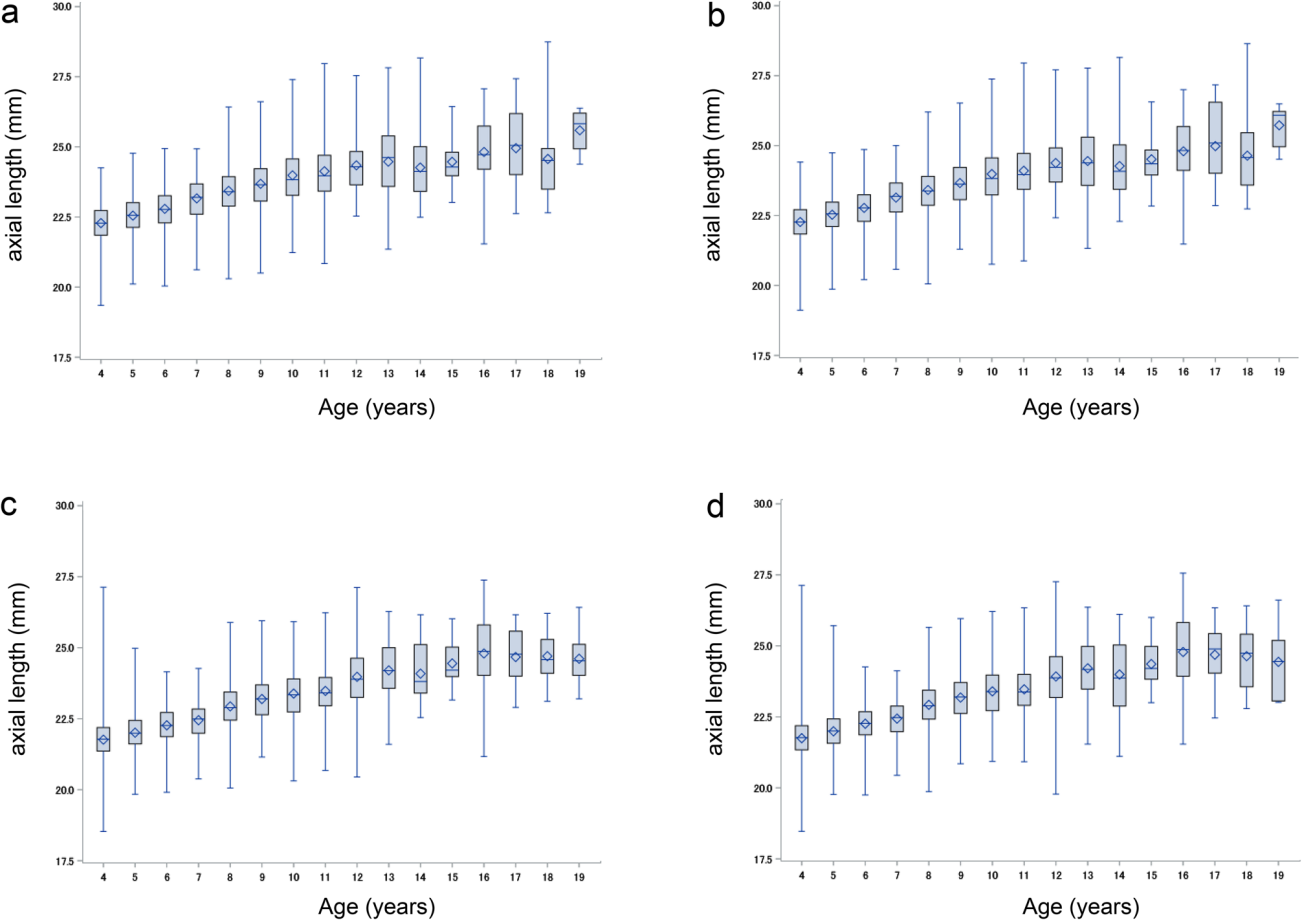
Box plot of axial length. The age-specific distribution of axial length by each eye and sex. a (boys; right eyes), b (boys; left eyes), c (girls; right eyes), d (girls; left eyes)

Among 33,189 eligible participants, AL measurements were available for 14,482 (43.63%), 7,025 (48.51%) of whom were girls (Fig.1, Table 1). The mean age was 6.3 ± 2.7 years (median 5 years, range 4-19 years). The mean AL was 22.65 ± 1.06 mm (range 18.53-28.64 mm) OD and 22.63 ± 1.07 mm (range 18.47-28.64 mm) OS. Figure 2 shows the age-specific distribution of AL in right and left eyes and by sex. Table 2 shows the mean, standard deviation, and maximum and minimum values for AL by age and sex. The average AL of boys was longer than the average AL of girls from ages 4 to 19, except at age 18. Notably, boys had a significantly longer AL than girls between the ages of 4 and 12. The right-eye AL was significantly longer than the left-eye AL at ages 4, 5, and 8 (p < 0.001, p < 0.001, and p = 0.0016, respectively).

**Table 1:** Basic characteristics

Parameter	Mean or number (SD or %)
Age (years)	6.3 (2.7)
Sex (girls, %)	7025 (48.5)
Height (cm)	116.9 (17.1)
Weight (kg)	23.0 (10.3)
Right axial length (mm)	22.6 (1.1)
Left axial length (mm)	22.6 (1.1)

**Table 2:** Distribution of axial length (AL) by age and sex

A. Right eyes																
Age	Total AL					Boys’ AL					Girls’ AL					*p*-value
	n	mean	SD	min	max	n	mean	SD	min	max	n	mean	SD	min	max	Boys vs. Girls
4years	5749	22.03	0.70	18.53	27.13	2968	22.28	0.65	19.35	24.25	2781	21.76	0.65	18.53	27.13	<0.0001
5years	2157	22.28	0.70	19.84	24.98	1094	22.55	0.65	20.11	24.77	1063	22.01	0.63	19.84	24.98	<0.0001
6years	993	22.53	0.75	19.91	24.94	508	22.79	0.73	20.04	24.94	485	22.27	0.66	19.91	24.15	<0.0001
7years	607	22.81	0.79	20.38	24.93	305	23.16	0.73	20.62	24.93	302	22.45	0.68	20.38	24.27	<0.0001
8years	2283	23.20	0.85	20.06	26.42	1218	23.43	0.82	20.30	26.42	1065	22.93	0.81	20.06	25.89	<0.0001
9 years	974	23.45	0.88	20.50	26.61	513	23.68	0.86	20.50	26.61	461	23.19	0.84	21.15	25.95	<0.0001
10 years	704	23.68	1.03	20.31	27.40	350	23.99	1.05	21.23	27.40	354	23.39	0.92	20.31	25.92	<0.0001
11 years	390	23.80	1.05	20.68	27.97	194	24.13	1.04	20.84	27.97	196	23.48	0.96	20.68	26.23	<0.0001
12 years	182	24.17	1.03	20.45	27.54	96	24.34	0.93	22.53	27.54	86	23.98	1.10	20.45	27.12	0.02
13 years	116	24.33	1.11	21.35	27.82	57	24.47	1.22	21.35	27.82	59	24.20	0.99	21.60	26.27	0.2
14 years	71	24.18	1.12	22.49	28.17	36	24.26	1.21	22.49	28.17	35	24.09	1.02	22.54	26.16	0.5
15 years	35	24.46	0.86	23.02	26.44	15	24.47	0.94	23.02	26.44	20	24.45	0.82	23.16	26.02	0.95
16 years	129	24.81	1.21	21.17	27.38	64	24.82	1.11	21.54	27.07	65	24.80	1.32	21.17	27.38	0.9
17 years	50	24.78	1.18	22.62	27.43	19	24.95	1.45	22.62	27.43	31	24.67	1.00	22.90	26.16	0.4
18 years	29	24.64	1.22	22.65	28.74	14	24.57	1.53	22.65	28.74	15	24.70	0.89	23.11	26.21	0.8
19 years	13	25.06	1.01	23.20	26.42	6	25.59	0.79	24.38	26.38	7	24.61	1.01	23.20	26.42	0.08

B. Left eyes																
Age	Total AL					Boys’ AL					Girls’ AL					*p*-value
	n	mean	SD	min	max	n	mean	SD	min	max	n	mean	SD	min	max	Boys vs. Girls
4 years	5749	22.02	0.71	18.47	27.13	2968	22.27	0.65	19.12	24.41	2781	21.75	0.66	18.47	27.13	<0.0001
5 years	2157	22.26	0.71	19.77	25.71	1094	22.53	0.65	19.87	24.74	1063	21.99	0.65	19.77	25.71	<0.0001
6 years	993	22.52	0.74	19.75	24.86	508	22.77	0.72	20.21	24.86	485	22.26	0.67	19.75	24.26	<0.0001
7 years	607	22.79	0.80	20.44	25.00	305	23.14	0.75	20.58	25.00	302	22.44	0.70	20.44	24.12	<0.0001
8 years	2283	23.19	0.85	19.87	26.20	1218	23.41	0.82	20.06	26.20	1065	22.92	0.81	19.87	25.65	<0.0001
9 years	974	23.43	0.88	20.85	26.52	513	23.66	0.85	21.30	26.52	461	23.18	0.85	20.85	25.96	<0.0001
10 years	704	23.69	1.04	20.76	27.38	350	23.98	1.05	20.76	27.38	354	23.40	0.93	20.93	26.21	<0.0001
11 years	390	23.78	1.05	20.88	27.95	194	24.10	1.02	20.88	27.95	196	23.47	0.98	20.92	26.34	<0.0001
12 years	182	24.16	1.08	19.78	27.71	96	24.38	0.98	22.42	27.71	86	23.92	1.13	19.78	27.26	0.004
13 years	116	24.33	1.11	21.33	27.77	57	24.45	1.22	21.33	27.77	59	24.21	1.00	21.54	26.36	0.3
14 years	71	24.13	1.16	21.11	28.15	36	24.27	1.18	22.29	28.15	35	23.99	1.14	21.11	26.11	0.3
15 years	35	24.43	0.91	22.84	26.56	15	24.51	0.95	22.84	26.56	20	24.36	0.90	23.00	26.00	0.6
16 years	129	24.79	1.20	21.48	27.56	64	24.80	1.08	21.48	27.00	65	24.78	1.32	21.54	27.56	0.95
17 years	50	24.80	1.19	22.46	27.17	19	24.98	1.44	22.86	27.17	31	24.69	1.03	22.46	26.34	0.4
18 years	29	24.64	1.26	22.74	28.64	14	24.64	1.49	22.74	28.64	15	24.64	1.05	22.79	26.41	0.99
19 years	13	25.03	1.23	23.01	26.61	6	25.73	0.79	24.51	26.49	7	24.44	1.26	23.01	26.61	0.054

*P*-values were obtained with t-tests for evaluating differences in AL between boys and girls. AL, axial length; SD, standard deviation

**Fig. 3:**
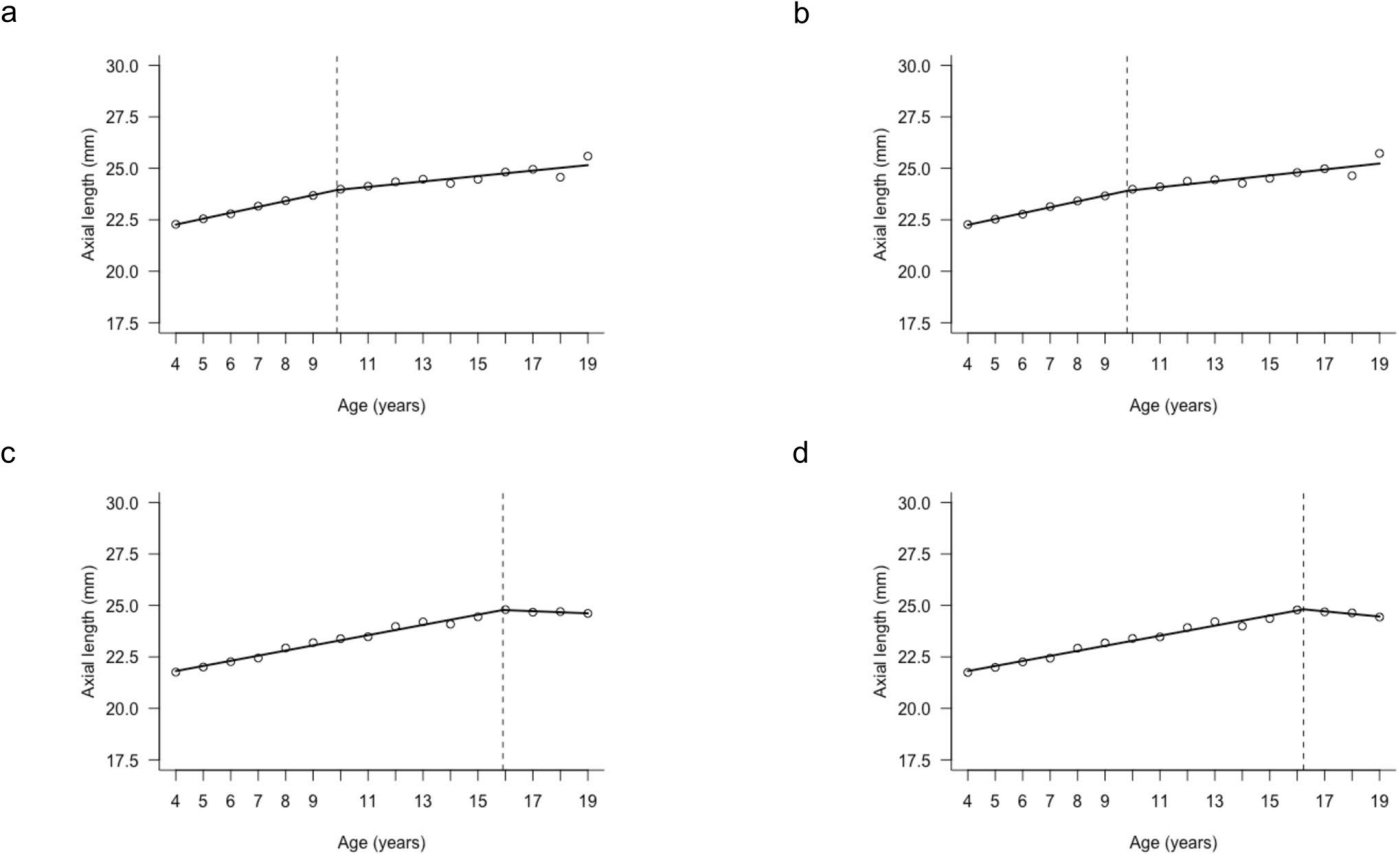
Segmented regression analysis of axial length according to age. Each point represents the mean axial length at each age. The line shows the fitted segmented regression line, and the dashed line indicates the estimated breakpoint between growth phases. a (boys; right eyes), b (boys; left eyes), c (girls; right eyes), d (girls; left eyes)

Segmented regression analysis showed that the break point in the mean right-eye AL by age occurred at 11.73 years. The slope before the break point was β = 0.27 (95% confidence interval [CI]: 0.23-0.30), while the slope after the break point decreased to β = 0.12 (95% CI: 0.08-0.16). For left-eye AL, the break point was identified at 11.67 years, with the slope before the break point being β = 0.27 (95% CI: 0.23-0.31) and the slope after the break point being β = 0.12 (95% CI: 0.08-0.16). These findings indicate that AL tends to increase with age up to around 12 years, after which the rate of elongation slows down. By sex, the break point in the mean right-eye AL by age occurred at 9.87 years in boys and at 15.91 years in girls. In boys, the slope before the break point was β = 0.29 (95% CI: 0.18-0.40) and after the break point, it was β = 0.13 (95% CI: 0.08-0.18). In girls, the slope before the break point was β = 0.25 (95% CI: 0.23-0.27), while the slope after the break point was β = -0.05 (95% CI: -0.17 to 0.07). For left-eye AL, the break point occurred at 9.80 years in boys and at 16.23 years in girls. In boys, the slope before the break point was β = 0.28 (95% CI: 0.17-0.40), and after the break point it was β = 0.14 (95% CI: 0.09-0.20). In girls, the slope before the break point was β = 0.25 (95% CI: 0.22-0.27), and after the break point it was β = -0.12 (95% CI: -0.34-0.09) (Fig. 3).

An AL ≥ 24.5 mm was rarely observed in children under 7 years of age—0.02% at age 4, 0.19% at age 5, 0.60% at age 6, and 1.81% at age 7. However, it became more common in children aged 8 and older, increasing from 7.67% at age 8 to 37.91% at age 12. Among participants aged 13 years and older, more than half had an axial length of ≥24.5 mm. The proportion of children with AL ≥ 26 mm increased between the ages of 10 and 12, rising from 2.7% to 6.6%. Among participants aged 13 years and older, 12.9 % had AL of ≥26 mm. When comparing boys and girls, the proportion of children with AL ≥ 24.5mm was significantly higher in boys aged 7 to 10 years, and the proportion of children with AL ≥ 26mm was significantly higher in boys aged 10 to 11 years. After the age of 12, no sex differences were observed in the prevalence of LAL (Table 3).

**Table 3:** The proportion of children with an AL of 24.5 mm or more or 26 mm or more in either the right or left eye

	AL ≥ 24.5 mm				AL ≥ 26 mm			
Age	All	Boys	Girls	*p*	All	Boys	Girls	*p*
Total	871 (6.0)	569 (7.6)	302 (4.3)	<0.0001	111 (0.8)	76 (1.0)	35 (0.5)	0.0004
4 years	1 (0.02)	0 (0)	1 (0.04)	0.5	1 (0.02)	0 (0)	1 (0.04)	0.5
5 years	4 (0.2)	3 (0.3)	1 (0.09)	0.6	0 (0)	0 (0)	0 (0)	-
6 years	6 (0.6)	6 (1.2)	0 (0)	0.03	0 (0)	0 (0)	0 (0)	-
7 years	11 (1.8)	11 (3.6)	0 (0)	0.009	0 (0)	0 (0)	0 (0)	-
8 years	175 (7.7)	136 (11.2)	39 (3.7)	<0.0001	3 (0.1)	3 (0.3)	0 (0)	0.3
9 years	126 (12.9)	90 (17.5)	36 (7.8)	<0.0001	4 (0.4)	4 (0.8)	0 (0)	0.1
10 years	154 (21.9)	103 (29.4)	51 (14.4)	<0.0001	19 (2.7)	17 (4.9)	2 (0.6)	0.0003
11 years	94 (24.1)	64 (33.0)	30 (15.3)	0.3	15 (3.4)	12 (6.2)	3 (1.5)	0.02
12 years	69 (37.9)	40 (41.7)	29 (33.7)	0.6	12 (6.6)	8 (8.3)	4 (4.7)	0.4
13 years	55 (47.4)	29 (50.9)	26 (44.1)	0.6	9 (7.8)	7 (12.3)	2 (3.4)	0.09
14 years	27 (38.0)	15 (41.7)	12 (34.3)	0.5	4 (5.6)	3 (8.3)	1 (2.9)	0.6
15 years	14 (40.0)	7 (46.7)	7 (35)	0.6	2 (5.7)	1 (6.7)	1 (5.0)	1
16 years	81 (62.8)	42 (65.6)	39 (60)	0.6	26 (20.2)	11 (17.2)	15 (23.1)	0.5
17 years	29 (58.0)	10 (52.6)	19 (61.3)	0.6	8 (16.0)	5 (26.3)	3 (9.7)	0.2
18 years	15 (51.7)	7 (50.0)	8 (53.3)	1	3 (10.4)	1 (7.1)	2 (13.3)	1
19 years	10 (76.9)	6 (100.0)	4 (57.1)	0.2	5 (38.5)	4 (66.7)	1 (14.3)	0.1
13-19years	231 (52.1)	116 (55.0)	115 (49.6)	0.3	57 (12.9)	32 (15.2)	25 (10.8)	0.2

AL, axial length

The proportion of children with AL ≥ 24.5 mm or AL ≥ 26 mm in boys and girls was compared with Fisher’s exact test

## Discussion

We analyzed data from 14,482 individuals from the TMM BirThree Cohort Study aged 4 to 19 years to determine the distribution of their AL. Of note, the data from 8,889 individuals aged 4 to 6 years was particularly valuable, as such a large data set has not been previously reported on a global scale. A segmented regression analysis identified that the break point for mean AL was approximately at the age of 12 years. Before this age, AL tended to elongate (β = 0.27), while after it, the elongation rate slowed (β = 0.12). Furthermore, when analyzed separately by sex, the break point was identified as occurring at approximately 10 years of age in boys and 16 years in girls. AL elongation was steeper in boys (β = 0.29) than in girls (β = 0.25). We determined the proportion by age of individuals with an AL ≥ 24.5 mm and ≥ 26 mm, which began to increase from ages 8 and 10 respectively. The proportion of individuals with LAL was higher among boys than girls between the ages of 6 and 11. After age 12, no significant sex differences were observed in the proportion of individuals with LAL.

The AL of the 4- to 6-year-old children was 22.14 ± 0.73 mm (among 8,899 children) in our study, significantly lower (p < 0.001) than the 22.35 mm ± 0.67 mm (among 457 children) reported by Matsumura et al. in Japan [16]. Chen et al. report that among East Asians and non-East Asians aged 4.0 to 6.9 years, AL was 22.40 (95% CI: 22.32-22.48) mm and 22.31 (95% CI: 22.16-22.47) mm, respectively [22]. Compared to these reports, we found that the AL of 4- to 6-year-old children was shorter than non-East Asians or East Asians. AL at ages 4 to 6 is thought to be influenced by genetic factors such as ethnicity and body size. However, even within the same Japanese population, significant differences in AL have been observed. One possible explanation is that the study by Matsumura et al. was conducted among preschool children in urban Kanagawa Prefecture, whereas the present study was conducted in Miyagi and Iwate prefectures, which are relatively rural. This environmental difference may have contributed to the variation in AL [23, 24]. Moreover, we compared mean, median, and quartile AL at 6, 9, and 15 years old with values for European children reported by Tideman et al. [11], as well as with values for Chinese children reported by Sans Diez et al. [12]. As shown in the Supplemental Table, we found significant differences in mean AL among the 3 groups at ages 6 and 9. However, at age 15, no significant difference was found between the Japanese and Chinese groups. Among the 3 groups, the Chinese participants had the longest AL, followed by the Japanese and the European children at ages 6 and 9. At age 15, AL in the Japanese and Chinese groups was similar, and both were longer than the European group. The median AL of the European children aligned with the 25th percentile of AL in the Japanese and Chinese distributions. Differences in AL among ethnic groups are observed at age 6, suggesting the influence of genetic factors [25]. By age 15, the AL of Chinese and Japanese participants had become comparable, indicating that lifestyle and environmental factors may also play a role [25].

The segmented regression analysis of the slope representing the speed of elongation showed that the break point for AL by age occurred at approximately 12 years. Before this age, the AL tended to elongate (β = 0.27), while after this age, the elongation rate slowed (β = 0.12). A review by Chen Y. et al. found that children’s AL grew at a rate of 0.3 to 0.4 mm per year between the ages of 3 and 5 [22], and Chen D.Z. et al. report that AL elongated at an average length of 0.80 mm at the ages of 3 to 6 years [26]. Similar to our results, a report by Fabian et al. targeting emmetropic eyes, found that the rate of AL elongation after the age of 12 was less than the reproducibility value (± 0.04 mm) of the AL measuring device [27].

In our data, the average AL of boys was longer than the average AL of girls from 4 to 19 years of age, except at the age of 18 years. Boys had significantly longer AL than girls between the ages of 4 and 12. Previous reports show that among adults, men have a longer AL than women [19, 28]. One study reports that there was a significant difference in mean AL between men and women among both those with myopia (p < 0.001) and those with hyperopia (p = 0.025) [29]. This is also supported by another study [30]. In a previous report on sex differences in AL in children under 7 years of age, both in children aged 0.0 to 1.9 years and in 2.0 to 3.9 years, AL was not significantly different between boys and girls (p = 0.85 and 0.37, respectively). In older children (4.0-6.9 years), boys were reported to have a longer AL than girls (boys: 22.49 mm, 95% CI 22.34-22.64, girls: 21.99 mm, 95% CI 21.82-22.15; p < 0.001) [22]. Recent studies report that it is possible to determine sex from fundus photographs in adults [31, 32], and Yamashita et al. note that boys with more masculine features in the fundus tend to have significantly longer ALs [33]. It is reported that the accuracy of sex identification from fundus images was low in younger elementary-school-aged children but significantly increased with age [34]. Specifically, the accuracy was recorded at 56.3% at 8.5 years, 46.1% at 9.5 years, 65.5% at 10.5 years, and 73.1% at 11.5 years [35]. It is not clear why boys have a longer AL than girls. One possible explanation could be that boys generally have a larger body size, greater height, and larger ocular volume [15, 28]. Segmented regression analysis revealed a difference in the break point of AL elongation speed between boys and girls. In boys, AL elongation was steep and tended to slow by the age of 10, while in girls, AL continued to elongate gradually until the age of 16. The reason for this sex difference in AL elongation patterns remains unclear, but differences in choroidal blood flow and hormones are considered possible factors. The choroid plays an important role in the regulation of eye growth and the development of myopia [36, 37]. Choroidal vascularity and choriocapillaris blood perfusion are lower in the more myopic eyes [38]. Choroidal circulation changes follow the menstrual cycle [39]. This may underlie the difference in break points for mean AL between the sexes and the tendency for AL to continue to extend in girls. To understand the reasons for sex differences in AL, further investigation is needed into ocular morphology as well as ocular volume, height, blood perfusion, and hormone levels.

In this study, we found that the right-eye AL was longer than the left-eye AL in participants aged 4, 5, and 8 years, a similar finding as previous studies. Several reports find that the right eye is more myopic [19, 40, 41]. The reason why the right eye is more predisposed to myopia is unknown, but it may be related to differences in the blood supply to the eye or hand dominance. Mansour et al. report that 93% of subjects in their study were right-handed and that SE was significantly less in their right eyes than their left eyes, but that there was no correlation between hand dominance and SE [41]. The right common carotid artery may have lower blood flow because of anatomical differences [42], which may be related to lower choroidal blood flow and a longer AL in the right eye [36, 38].

In this study, we calculated the age-specific proportions of participants with AL ≥ 24.5 mm and ≥ 26 mm, defined as LAL, which were considered indicative of suspected myopia. Among children aged 4 to 7 years, the prevalence of participants with AL ≥ 24.5 mm was quite low; however, a few children with longer AL were still observed, and these individuals were highly suggestive of having already reached the stage of high myopia. The proportions AL ≥ 24.5mm began to increase from age 8, continued to rise with age, and reached approximately half of the population by age 13 and older. The proportion of children with AL ≥ 26 mm, associated with significantly increased risk of serious complications later in life, increased particularly between ages 10 and 12, and about 10% were observed among those aged 13 years and older. When comparing boys and girls, we found that the proportion with LAL was significantly higher among boys aged 7 to 11 years. After the age of 12, no gender differences were observed in the prevalence of LAL. These results suggest that 8 years may be the age at which the onset of LAL occurs, and that many children are at risk of developing high myopia by the time they reach age 10. The higher prevalence of LAL in boys compared to girls before age 11, and the absence of sex differences after age 12, may be attributed to differences in the rate of axial elongation, as suggested by the results of the segmented regression analysis. According to a report by the International Myopia Institute [5], myopia is less common in children younger than 6 years, and even in East Asia and Singapore, where the prevalence of adult myopia is high, most studies show that myopia is less common in children younger than 6 years; the prevalence has been shown to be less than 5% [43–46]. According to a survey of myopia by Matsumura et al. based on an auto reflex meter, myopia increased from the ages of 7 to 8 in Japan [16]. Our results are similar to previous reports. Chua et al. observed schoolchildren aged 7 to 9 until age 11 and show that the age at onset of myopia was the strongest predictor of high myopia [47]. With high myopia, 85% of children were likely to have developed myopia before age 7. Olavi et al. report that 32% of children who received their first prescription for eyeglasses between the ages of 8.8 and 12.8 years developed high myopia in adulthood [48]. These reports and our current data indicate that AL before age 8 plays an important role in predicting future myopia.

As this study encompassed data collected before, during, and after the COVID-19 lockdowns in Japan—periods during which excessive AL elongation among preschool children has been widely reported [49–52]. The potential influence of the COVID-19 lockdown on AL should also be considered. When comparing age-specific AL before and after the nationwide lockdown in Japan, which took place around April 2020, a statistically significant difference was observed only in the right eyes of 13-year-old participants, whereas no significant differences were found in other age groups. In 13-year-olds, the mean AL before the lockdown was 24.13 ± 1.05 mm (n = 81), and after the lockdown it was 24.81 ± 1.12mm (n = 35), with a *p*-value of 0.002.

There are concerns that many children are at risk of visual impairment in the future. Since the progression of myopia is irreversible, it is important to detect high-risk cases early and link them to treatment. The results of this study can be used as a reference for determining standard values for AL at each age and sex and predicting future myopia. It may be useful for designing appropriate myopia-prevention programs [53, 54]. Early onset and prolonged axial elongation may increase the risk of developing high myopia in the future. Therefore, a longitudinal approach that includes follow-up over time will be necessary. We plan to continue to observe this cohort over the long term, and we expect to obtain further valuable findings.

The present study has several limitations. Refractive power directly indicates the degree of myopia, but in this study, refractive test results were not included in the analysis. In addition to AL, various factors, such as the corneal radius of curvature, anterior chamber depth, and crystalline lens thickness determine the refractive power of the eye, but these test results were not included in the results of this study. Due to the characteristics of the cohort enrollment, measurements were more frequently obtained at ages 4 and 8. The data for children aged > 8 years are derived only from sibling category of the TMM BirThree Cohort Study. A total of 2,801 participants provided two measurements. Therefore, the age distribution is skewed, which may have influenced the analysis. Potential biases due to the unbalanced age distribution include reduced precision of estimates in the older age group because of the small sample size, distortion of age-related trends caused by the over-representation of younger age group, and limited generalizability of the findings to older children and adolescents. In addition, subtle changes in AL could remain undetected owing to the insufficient number of cases in that age range. Follow-up AL measurements have not yet been performed with the participants and therefore changes associated with growth cannot be determined. Furthermore, the number of participants in older age groups was small; therefore, the data from these groups should be interpreted carefully.

This study was the first large-scale AL survey in Japan and reveals the age and sex related distribution of AL and the proportion with myopia among Japanese children and adolescents aged 4 to 19 years.

## Supplementary Information

Below is the link to the electronic supplementary material.

Supplementary file1 (DOCX 19 KB)

## Data Availability

The data that support the findings of this study are available from the corresponding author upon reasonable request.
