# Supplementary material for: Distribution of axial length in Japanese children and adolescents aged 4 to 19 years
Source: Jpn J Ophthalmol. 2026 Jan 19;70(3):502–11. doi: 10.1007/s10384-026-01328-1 (PMC13226453; doi:10.1007/s10384-026-01328-1)
Supplement: Supplementary file 1 — Supplementary file1 (DOCX 19 KB) [file 10384_2026_1328_MOESM1_ESM.docx]

Supplemental Table. Percentiles of axial length (AL) in 6-,9-, and 15-year-old European, Chinese, and Japanese children of both sexes

|  |  | Boys | | |  | Girls | | |
| --- | --- | --- | --- | --- | --- | --- | --- | --- |
|  |  | European* | Chinese** | Japanese |  | European* | Chinese** | Japanese |
|  |  |  |  | (current study) |  |  |  | (current study) |
| 6 years | n | 3033 | 500 | 508 |  | 3051 | 474 | 485 |
|  | Mean ± SD | 22.63±0.73 | 23.03 ± 0.82 | 22.79 ± 0.73 |  | 22.09±0.70 | 22.56±0.78 | 22.27 ± 0.66 |
|  | 25 Percentiles | 22.14 | 22.55 | 22.29 |  | 21.66 | 22.03 | 21.87 |
|  | 50 Percentiles | 22.59 | 22.99 | 22.77 |  | 22.06 | 22.54 | 22.27 |
|  | 75 Percentiles | 23.01 | 23.50 | 23.26 |  | 22.49 | 23.04 | 22.68 |
| 9 years | n | 2617 | 912 | 513 |  | 2679 | 917 | 461 |
|  | Mean ± SD | 23.36±0.82 | 24.26 ± 0.92 | 23.68 ± 0.86 |  | 22.84±0.78 | 23.73±0.89 | 23.19 ± 0.84 |
|  | 25 Percentiles | 22.83 | 23.7 | 23.07 |  | 22.33 | 23.16 | 22.62 |
|  | 50 Percentiles | 23.31 | 24.32 | 23.65 |  | 22.79 | 23.72 | 23.20 |
|  | 75 Percentiles | 23.79 | 24.89 | 24.22 |  | 23.25 | 24.31 | 23.71 |
| 15 years | n | 1167 | 127 | 15 |  | 1328 | 110 | 20 |
|  | Mean ± SD | 23.68±0.88 | 25.15 ± 1.28 | 24.47 ± 0.94 |  | 23.18±0.84 | 24.50±1.15 | 24.44 ± 0.82 |
|  | 25 Percentiles | 23.17 | 24.39 | 23.42 |  | 22.68 | 23.83 | 23.83 |
|  | 50 Percentiles | 23.65 | 25.01 | 24.28 |  | 23.15 | 24.37 | 24.21 |
|  | 75 Percentiles | 24.21 | 25.80 | 25.01 |  | 23.65 | 25.20 | 24.98 |

SD, standard deviation.

*Tideman JW, Snabel MC, Tedja MS, van Rijn GA, Wong KT, Kuijpers RW, Vingerling JR, Hofman A, Buitendijk GH, Keunen JE, Boon CJ, Geerards AJ, Luyten GP, Verhoeven VJ, Klaver CC. Association of Axial Length with Risk of Uncorrectable Visual Impairment for Europeans With Myopia. JAMA Ophthalmol. 2016;134(12):1355-63.

**Sanz Diez P, Yang LH, Lu MX, Wahl S, Ohlendorf A. Growth curves of myopia-related parameters to clinically monitor the refractive development in Chinese schoolchildren. Graefes Arch Clin Exp Ophthalmol. 2019;257(5):1045-53.

The mean AL in the three groups was compared using Welch’s ANOVA, followed by pairwise t-tests with Bonferroni correction. At ages 6 and 9, significant differences were observed among all three groups (*p* < 0.001). At age 15, no significant difference was found between the Japanese and Chinese groups (*p* = 0.99 in girls, *p* = 0.058 in boys), whereas significant differences were observed between the Japanese and European groups (*p* < 0.001 in girls, *p* = 0.017 in boys) and between the Chinese and European groups (*p* < 0.001)
